# Lipoic Acid-Functionalized Hexanuclear Manganese(III) Nanomagnets Suitable for Surface Grafting

**DOI:** 10.3390/ijms24108645

**Published:** 2023-05-12

**Authors:** Marta Orts-Arroyo, Carlos Rojas-Dotti, Nicolás Moliner, José Martínez-Lillo

**Affiliations:** Departament de Química Inorgànica, Instituto de Ciencia Molecular (ICMol), Universitat de València, c/Catedrático José Beltrán 2, 46980 Paterna, Spain; marta.orts-arroyo@uv.es (M.O.-A.); carlos.rojas@uv.es (C.R.-D.); fernando.moliner@uv.es (N.M.)

**Keywords:** manganese, lipoic acid, salicylamidoxime, crystal structure, magnetic susceptibility, single-molecule magnet

## Abstract

Highly anisotropic single-molecule magnets (SMMs) have attracted much interest in the field of molecular magnetism because of their spin features and potential technological applications. Additionally, a great effort has been devoted to the functionalization of such molecule-based systems which are made with ligands containing functional groups suitable to connect SMMs to junction devices or to perform their grafting on surfaces of different substrates. We have synthesized and characterized two lipoic acid-functionalized and oxime-based Mn(III) compounds, of formula [Mn_6_(μ_3_-O)_2_(H_2_N-sao)_6_(lip)_2_(MeOH)_6_][Mn_6_(μ_3_-O)_2_(H_2_N-sao)_6_(cnph)_2_(MeOH)_6_]}·10MeOH (**1**) and [Mn_6_(μ_3_-O)_2_(H_2_N-sao)_6_(lip)_2_(EtOH)_6_]·EtOH·2H_2_O (**2**) [H_2_N-saoH_2_ = salicylamidoxime, lip = lipoate anion, cnph = 2-cyanophenolate anion]. Compound **1** crystallizes in the space group *P*ī of the triclinic system and **2** crystallizes in the space group C2/c of the monoclinic system. In the crystal, neighboring Mn_6_ entities are linked using non-coordinating solvent molecules, which are H-bonded to N atoms of –NH_2_ groups of amidoxime ligand. In addition, Hirshfeld surfaces of **1** and **2** were calculated to study the variety of intermolecular interactions and the different levels of importance that take place in their crystal lattice; this type of computed study is the first time performed on Mn_6_ complexes. The study of the magnetic properties of **1** and **2** through dc magnetic susceptibility measurements reveals the coexistence of ferromagnetic and antiferromagnetic exchange couplings between the Mn(III) metal ions in both compounds, the latter being the predominant magnetic interaction. A spin S = 4 value of the ground state was obtained using isotropic simulations of the experimental magnetic susceptibility data for both **1** and **2**. Ac magnetic susceptibility measurements show features typical of slow relaxation of the magnetization in **1** and **2**, which indicate that SMM behavior takes place in both compounds.

## 1. Introduction

Single-molecule magnets (SMMs) are molecules that can act as nanomagnets at the molecular level and in low temperatures; these compounds are suitable candidates to investigate potential technological applications, some of which can be high-density data storage, quantum computing and molecular refrigeration [1,2,3,4,5,6]. Hence, the singular magnetic properties that these compounds exhibit can also be investigated in functional devices, mainly in the research fields of molecular spintronics and molecular electronics [7,8,9,10]. The self-assembly of SMMs on surfaces and junction devices has been mainly reported for molecules functionalized with sulfur-based groups, which have allowed for the grafting of them onto adapted substrates of gold, silicon and nickel, thus generating new materials of technological interest [11,12,13,14].

The synthesis of hexanuclear Mn(III) complexes (Mn_6_) starting from Mn(II) salts and phenolic oximes, such as salicylaldoximes and salicylamidoximes and their derivative molecules, has been investigated during the last two decades and has proven to be a successful and reliable method for the preparation of Mn(III)-based SMMs, as all these discrete Mn_6_ molecules display this singular magnetic behavior [15,16,17,18,19,20,21,22,23,24,25,26,27].

Both experimental and theoretical studies performed on this family of oxime-based Mn_6_ SMMs have established that these compounds exhibit spin ground states (S) that vary from 4 to 12 and anisotropy energy barriers (U_eff_) ranging from 24 to 86 K. Interestingly, the magnetic coupling involving the Mn(III) metal ions varies according to the Mn–N–O–Mn set of atoms, which forms significant angles in oxime-based Mn_6_ complexes. Thus, a critical angle in which the global magnetic coupling among adjacent Mn(III) metal ions changes from antiferromagnetic to ferromagnetic has been found. This critical angle is approximately 27° for salicylamidoxime-based compounds and ca. 31° for salicylaldoxime-based complexes [18,21,24].

Some of these Mn_6_ molecules have been functionalized using thioester-based ligands and have been connected using the lift-off method to two nickel electrodes of an exposed-edge tunnel junction [28]. Transport studies performed at room temperature on this device have shown an increase in the current with the presence of this functionalized Mn_6_ complex [29,30]. Theoretical calculations performed on this type of molecule-based device have been carried out to investigate their molecular features and spin properties [30].

**Scheme 1:**
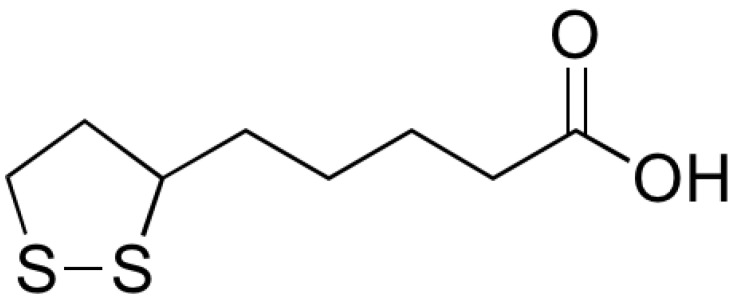
Molecular structure of the D,L-α-lipoic acid.

In the literature, there only exists an oxime-based Mn(III) nanomagnet obtained with lipoic acid. It is a chiral, decanuclear Mn(III) complex containing non-coordinated sulfur atoms (Scheme 1) which is suitable for surface grafting [31]. Hence, the investigation in the coordination chemistry of complexes based on lipoic acid is clearly underdeveloped, and it is very appealing to get new insights into this type of study.

Herein, we describe the preparation, structural characterization and investigation of the magnetic characteristics of two oxime-based Mn(III) compounds with the formula [Mn_6_(μ_3_-O)_2_(H_2_N-sao)_6_(lip)_2_(MeOH)_6_][Mn_6_(μ_3_-O)_2_(H_2_N-sao)_6_(cnph)_2_(MeOH)_6_]}·10MeOH (**1**) and [Mn_6_(μ_3_-O)_2_(H_2_N-sao)_6_(lip)_2_(EtOH)_6_]·EtOH·2H_2_O (**2**) [H_2_N-saoH_2_ = salicylamidoxime, Hlip = lipoic acid, cnph = 2-cyanophenolate], which constitute the second reported example of Mn(III)-based SMM containing lipoic acid.

## 2. Results and Discussion

### 2.1. Synthetic Procedure

By reacting salicylamidoxime and lipoic acid ligands together with a Mn(II) salt [MnCl_2_·4H_2_O for **1** and Mn(NO_3_)_2_·4H_2_O for **2**] in the presence of NEt_3_, a dark green solution was formed in MeOH and EtOH for **1** and **2**, respectively. The thus obtained solution provided a dark green microcrystalline solid in the case of the synthesis of **1** and dark green crystals in the case of **2**. The better yield covers the range of 55–70%. These fresh samples were the ones employed to perform the magnetism studies. In order to get crystals and to study the crystal structure of **1**, a concentrated solution of the microcrystalline solid obtained in methanol was layered with acetone, generating dark green crystals through solvent diffusion. Nevertheless, these crystals present two crystallographically independent Mn_6_ complexes; one of them contains lipoate ligand as compound 2, but the other is bearing 2-cyanophenolate ligand instead. It was generated by partial hydrolysis of salicylamidoxime during the re-crystallization process. The in situ formation of 2-cyanophenolate in syntheses of Mn_6_ complexes has been previously reported [24]. In any case, this synthetic procedure would be a straightforward method to add the lipoate ligand to the well-known family of Mn_6_ complexes.

### 2.2. Description of the Crystal Structures

**Figure 1:**
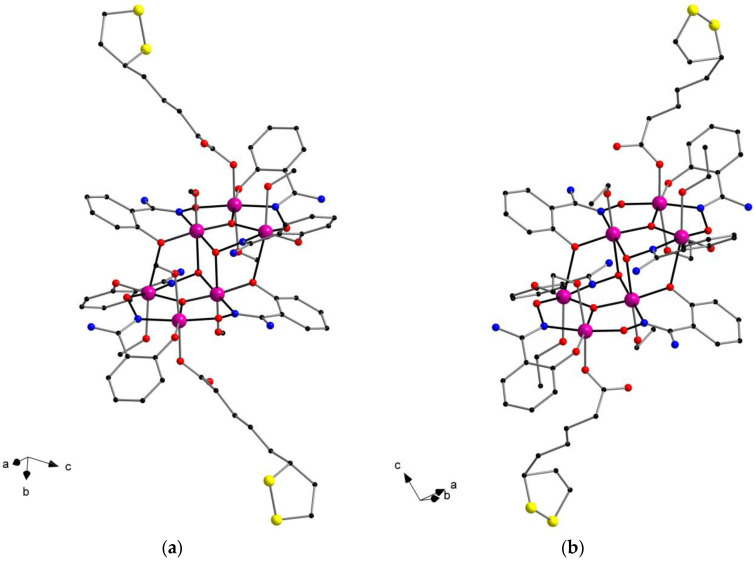
(**a**) Detail of the hexanuclear [Mn_6_(μ_3_-O)_2_(H_2_N-sao)_6_(lip)_2_(MeOH)_6_] complex in **1**; (**b**) detail of the hexanuclear [Mn_6_(μ_3_-O)_2_(H_2_N-sao)_6_(lip)_2_(EtOH)_6_] complex in **2**. H atoms and non-coordinating solvent molecules have been omitted. Color code: pink, Mn; yellow, S; red, O; blue, N; black, C.

The crystal structures of the reported complexes (**1** and **2**) are made up of neutral hexanuclear Mn(III) complexes, so-called Mn_6_ SMMs [15,16,17,18,19,20,21,22,23,24,25,26,27]. Compound **1** crystallizes in the space group Pī of the triclinic system and compound **2** crystallizes in the space group C2/c of the monoclinic system (see Table 1). The crystallization solvent molecules MeOH (**1**), H_2_O (**2**) and EtOH (**2**) are also present in the structures of these compounds. The characteristic Mn_6_ complexes found in **1** and **2** are shown in Figure 1. In this family of compounds, the core is formed by two {Mn_3_(μ_3_-O)} subunits, which display a connection between them through phenolate and oximate O atoms (Figure 1). Three Mn(III) metal ions of each {Mn_3_(μ_3_-O)} triangular subunit are mainly connected by three –N–O– bonds of salicylamidoxime molecules and an oxo group, which is located approximately in the plane containing the triangle in **1** and **2**.

In compound **1**, there are two crystallographically independent Mn_6_ complexes. The Mn(III) metal ions in the center of **1** and **2** show a metal environment very close to those of earlier published amidoxime-based Mn_6_ compounds [32,33], exhibiting elongated Oh geometries with axial axes pretty much located at 90° about the main plane of the triangular subunits. The Mn–N–O–Mn angles of the {Mn_3_(μ_3_-O)} triangular subunits are 43.7, 41.2 and 26.1° and 38.0, 36.2 and 26.3° for **1** and 42.9, 36.9 and 24.4° for **2**. These torsion angle values are very important to explain the magnetic properties of this type of Mn_6_ complexes [15,16,17,18,19,20,21,22,23,24,25,26,27,28,29,30,31,32,33]. In both compounds, the coordinated lipoate ligand is bonded to the Mn(3) ion and to its symmetry equivalent [Mn(3a); (a) = −x, −y, −z + 1 for **1** and (a) = −x + 1, −y + 1, −z + 1 for **2**], using the carboxylate group in a monodentate fashion, as previously reported for other carboxylate-based ligands in this family of complexes [15,16,17,18,19,20,21,22,23,24,25,26,27]. In both cases, the lipoate ligand exhibits average values of the S–S, C–S and C–C bond lengths of the dithiolane ring that are in agreement with those previously reported [31].

**Figure 2:**
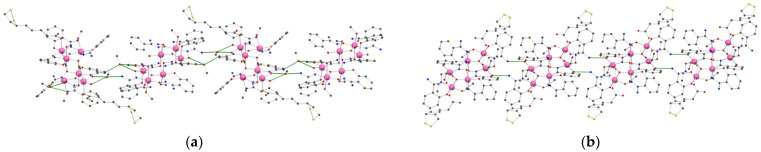
(**a**) One-dimensional motif generated by H-bonding interactions of homometallic [Mn_6_(μ_3_-O)_2_(H_2_N-sao)_6_(lip)_2_(MeOH)_6_] complexes along with non-coordinating solvent molecules in **1**; (**b**) view of the one-dimensional motif generated by H-bonding interactions of homometallic [Mn_6_(μ_3_-O)_2_(H_2_N-sao)_6_(lip)_2_(EtOH)_6_] complexes in **2**.

**Figure 3:**
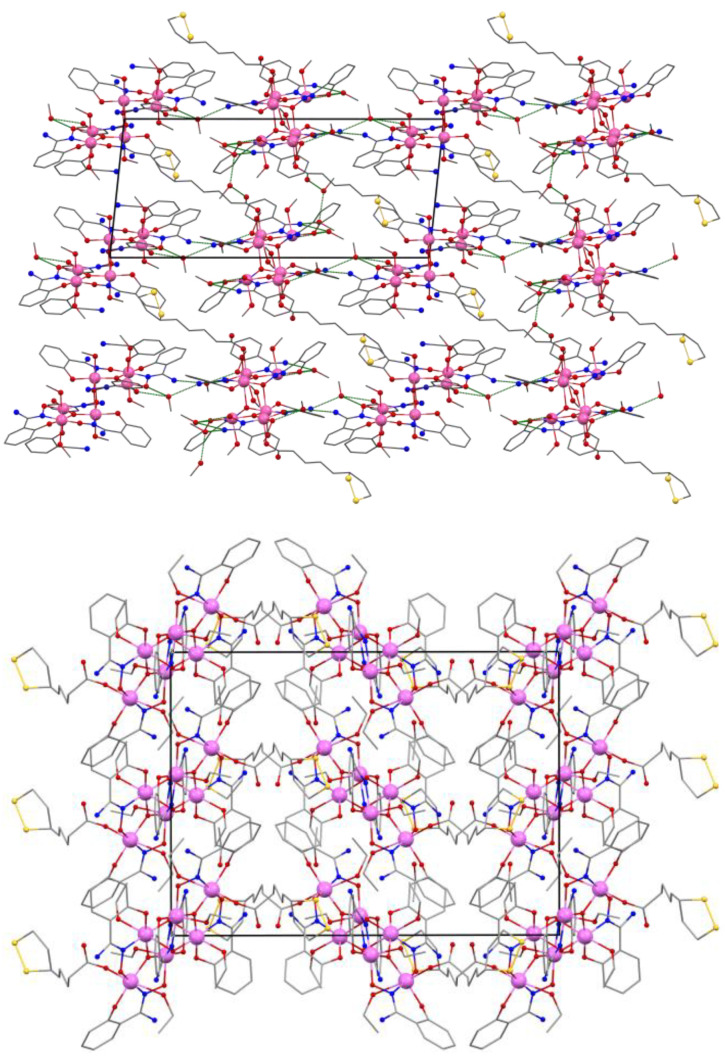
A fragment of the crystal arrangement of Mn_6_ complexes in **1** (**top**); a fragment of the packing of Mn_6_ complexes in **2** (**bottom**). In both cases, H atoms have been omitted.

In the molecules arrangement of **1**, the neutral Mn_6_ entities are linked through H-bonding interactions promoted by solvent MeOH molecules, oximate oxygen atoms and –NH_2_ groups of neighboring neutral Mn_6_ units via the N(9)···O(10)···O(24) pathway [N(9)···O(10) distance of ca. 2.89(1) Å and O(10)···O(24) distance of ca. 2.80(1) Å], thus generating a 1D motif based on Mn_6_ complexes (Figure 2). The shortest intermolecular S···S distance in **1** is approximately 11.44(1) Å [S(2)···S(1b) distance; (b) = x, y + 1, z]. Further intermolecular crystal interactions formed through S atoms of the dithiolane rings and –C-H groups of salicylamidoxime ligands of adjacent Mn_6_ complexes [S···C distances cover the 3.4(1)–3.8(1) Å range; (c) = −x + 1, −y + 1, −z + 1 and (d) = −x + 2, −y + 1, −z + 2] link the Mn_6_ pseudo-chains leading to a 2D structure of alternating Mn_6_ complexes in **1** (Figure 3).

In the molecules arrangement of **2**, adjacent discrete Mn_6_ entities are connected to each other through hydrogen bonds, which involve –NH_2_ groups and O atoms of phenolate groups of close salicylamidoxime ligands [N(2)···O(4b) separation of ca. 2.99(1) Å; (b) = −x + 3/2, −y + 3/2, −z + 1], leading to a 1D arrangement (Figure 2). Further H-bonded Mn_6_ molecules are linked by means of additional –NH_2_ and carboxylate groups of neighboring Mn_6_ entities [N(6)···O(11c) separation of approximately 3.05(1) Å; (c) = −x + 1, y, −z + 3/2], generating a 2D structure in **2** (Figure 3). The shortest intermolecular S···S distance in **2** is much more reduced than that found in **1** [S(1)···S(1d) separation of ca. 5.14(1) Å; (d) = −x + 2, −y + 1, −z + 2]. Finally, weak interactions between S atoms of the dithiolane rings and –C-H groups of salicylamidoxime ligands of neighboring complexes [S···C separations spanning the ca. 3.4(1)–3.8(1) Å range; (c) = −x + 1, −y + 1, −z + 1 and (d) = −x + 2, −y + 1, −z + 2] link the Mn_6_ pseudo-chains leading to a 2D structure of alternating Mn_6_ complexes in **2** (Figure 3).

### 2.3. Analysis of the Hirshfeld Surfaces

**Figure 4:**
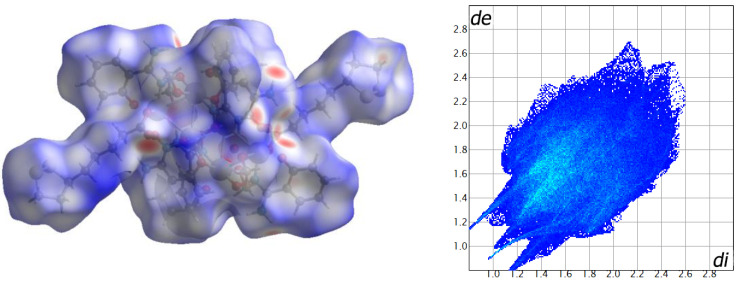
Hirshfeld surface mapped using d_norm_ expression for the hexanuclear Mn(III) complex in **1** (**left**); full fingerprint plot for complex **1** (**right**).

**Figure 5:**
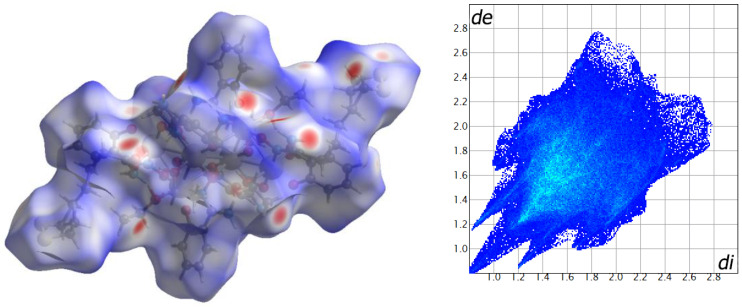
Hirshfeld surface mapped using d_norm_ expression for the hexanuclear Mn(III) complex in **2** (**left**); full fingerprint plot for complex **2** (**right**).

The intermolecular interactions involving the neutral Mn_6_ complexes of **1** and **2** were further studied through the CrystalExplorer program [34]. By mapping the distances of the 3D surface formed, through the nearest atom outside (d_e_) and inside (d_i_) of each part of the studied surface, and with a normalized contact distance (d_norm_) that overcomes some limitations generated by the atom size, this program allowed for the qualitative and quantitative study of the significant molecular contacts [34,35,36]. It is displayed with a set of colors. The red color is used for short contacts, white indicates contact interactions close to the van der Waals distances, and blue is assigned to long interactions. With all this information, a fingerprint is generated, which is a 2D plot outline of the involved molecular interactions [35,36,37,38]. The Hirshfeld surfaces for complexes **1** and **2** are the first ones reported for oxime-based Mn_6_ entities and are given in Figure 4 and Figure 5, respectively. The intermolecular O···H contacts formed among the -NH_2_ groups of salicylamidoxime ligands and solvent MeOH molecules, which connect the Mn_6_ complexes in **1**, are approximately 14% of the complete fingerprint plot (Figure 4). In addition, intermolecular weak S···H contacts involving S atoms of the dithiolane rings and –C-H groups of salicylamidoxime ligands of adjacent Mn_6_ entities cover ca. 6% of the graphic outline of complex **1** (Figure 4). In complex **2**, intermolecular O···H contacts generated by H-bonding interactions, which are formed between the -NH_2_ groups of salicylamidoxime ligands and carboxylate groups of neighboring Mn_6_ molecules, are close to 11% of the complete fingerprint plot (Figure 5) and, therefore, it is a somewhat lower percentage than that detected for O···H contacts in complex **1**. Finally, intermolecular S···H contacts are also observed in complex **2**, in this case displaying a higher percentage (ca. 12% of the complete fingerprint plot) than that shown in complex **1** (Figure 4 and Figure 5).

### 2.4. Study of the Magnetic Properties

**Figure 6:**
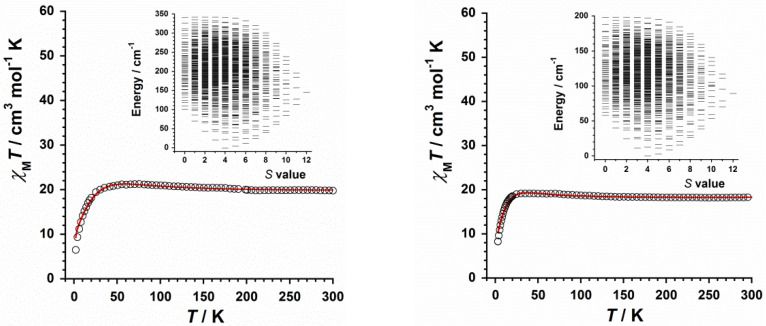
Thermal variation of the χ_M_T product for compounds **1** (**left**) and **2** (**right**). The solid red line indicates the fit of the experimental magnetic susceptibility values. The inset shows the plot of energy vs. spin value (S) obtained from the isotropic simulation of the magnetic susceptibility data for each compound.

Dc magnetic susceptibility studies were carried out on microcrystalline samples of both compounds in the temperature range from 300 to 1.9 K. An external magnetic field of H_dc_ = 0.5 T was employed. χ_M_T vs. T curves of **1** and **2** are shown in Figure 6. At T = 300 K, the initial χ_M_T values are approximately 19.2 (**1**) and 18.3 cm^3^mol^−1^K (**2**). These χ_M_T values are very close to that expected for six isolated Mn(III) metal ions, with S = 2 and g = 1.99 values for each metal ion, which are in accordance with those earlier published for amidoxime-based hexanuclear Mn(III) complexes [15,16,17,18,19,20,21,22,23,24,25,26,27]. With cooling, the χ_M_T value in complex **1** remains pretty much constant to 200 K, before χ_M_T slightly increases to reach a value of ca. 21.2 cm^3^mol^−1^K at 60 K. Afterwards, χ_M_T value decreases very fast to finish with a value of ca. 6.5 cm^3^mol^−1^K at 1.9 K (Figure 6). In complex **2**, χ_M_T value follows the Curie law by reducing the temperature to 150 K, before χ_M_T somewhat rises to obtain a maximum number of approximately 19.2 cm^3^mol^−1^K at 35 K. Finally, χ_M_T decreases very fast, reaching a final number of ca. 8.3 cm^3^mol^−1^K at 2 K (Figure 6). The decrease in the χ_M_T values displayed at low temperatures by **1** and **2** are likely due to the occurrence of molecular magnetic exchange and/or zero-field splitting (ZFS) consequence, which have been published earlier for close Mn_6_ systems [15,16,17,18,19,20,21,22,23,24,25,26,27].

The experimental magnetic susceptibility values of the *χ*_M_*T* vs. *T* curves of **1** and **2** were analyzed employing a 2*J* model which can be exemplified through the isotropic Hamiltonian shown in Equation (1), where *J*_1_ and *J*_2_ are the magnetic exchange constants for the manganese–manganese couplings assuming the pathway based on the Mn–N–O–Mn set of atoms in the Mn_6_ entity. *J*_1_ is assigned to higher Mn–N–O–Mn torsion angles and *J*_2_ to the lower ones (Figure S1). A term to account for the Zeeman result is also added to the final part of the Hamiltonian. The magnetic *g* parameter is the Landé factor for the involved metal ions. The best theoretical fit thus obtained gave the following parameters: *g* = 1.98, *J*_1_ = +1.95 cm^−1^ and *J*_2_ = −2.07 cm^−1^ for complex **1**, and *g* = 1.99, *J*_1_ = +1.78 cm^−1^ and *J*_2_ = −2.04 cm^−1^ for complex **2**. This theoretical 2*J* model reproduces very well the experimental magnetic susceptibility data in the reported temperature range (see solid red lines in Figure 6). The numbers computed for *J*_1_ and *J*_2_ are in line with the Mn–N–O–Mn angles obtained in the structures of both compounds and are in agreement with earlier reported values of similar Mn_6_ compounds [15,16,17,18,19,20,21,22,23,24,25,26,27,28]. In both compounds, the global antiferromagnetic interaction (associated with *J*_2_) is the main magnetic exchange between the implicated Mn(III) ions, even though both magnetic contributions are important in both **1** and **2**, as observed in their experimental curves (Figure 6), and both compounds exhibit at least a torsion angle value lower than the critical value of 27°, as indicated in Section 2.2 [21,22,23,24,25,26,27].

*Ĥ* = −2*J*_1_(Ŝ_1_·Ŝ_3_ + Ŝ_1_·Ŝ_3′_ + Ŝ_1_·Ŝ_1′_ + Ŝ_1′_·Ŝ_3_ + Ŝ_1′_·Ŝ_3′_) − 2*J*_2_(Ŝ_1_·Ŝ_2_ + Ŝ_2_·Ŝ_3_ + Ŝ_1′_·Ŝ_2′_ + Ŝ_2′_·Ŝ_3′_) + μ_B_*gH*Ŝ (1)

In general, in this family of Mn_6_ systems, the antiferromagnetic compounds display a spin S = 4 value, whereas the ferromagnetic compounds show a spin S = 12 value [15,16,17,18,19,20,21,22,23,24,25,26,27]. The reported *χ*_M_*T* vs. *T* plot for both **1** and **2** would be consistent with a global antiferromagnetic interaction and, a priori, a spin S = 4 value for the ground state would be expected for both **1** and **2**. In fact, the simulation of the magnetic susceptibility data of **1** and **2** generated the plots of the energy vs. spin value displayed in the respective insets in Figure 6, which was performed as in previous studies focused on Mn_6_ complexes [17,18,19,20,21]. In both compounds, the ground state spin value would be S = 4 and the first excited state would be S = 5, which is placed at 3.50 (**1**) and 2.97 cm^−1^ (**2**). In many other amidoxime-based Mn_6_ complexes with S = 4, which were previously studied, the first excited state was S = 3 [32,33]. These results match the S values obtained from the reduced magnetization data fits (Figures S2 and S3).

**Figure 7:**
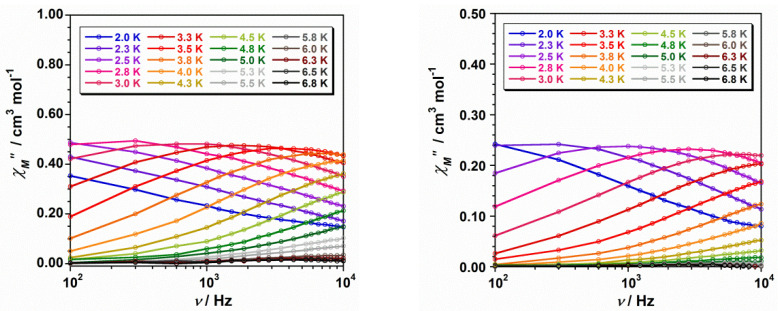
Frequency dependence of the out-of-phase ac magnetic susceptibility signals obtained with a magnetic field of H_dc_ = 0 G for **1** (**left**) and **2** (**right**).

**Figure 8:**
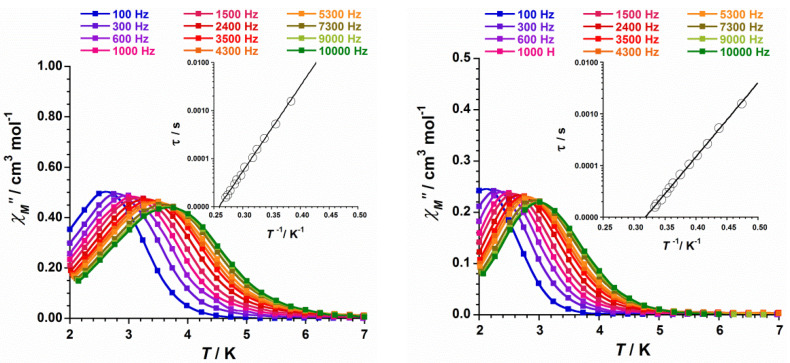
Out-of-phase ac magnetic susceptibility signals at several frequencies (100–10,000 Hz) obtained with a magnetic field H_dc_ = 0 G for **1** (**left**) and **2** (**right**). The inset displays the ln(τ) versus 1/T plot considering a theoretical fit with only an Orbach mechanism.

Ac magnetic susceptibility studies were carried out on microcrystalline samples of **1** and **2** in the temperature range from 7 to 2 K. An ac magnetic field of H_ac_ = 5.0 G oscillating at several frequency values (10^2^–10^4^ Hz range) was used. Slow relaxation of the magnetization is detected in **1** and **2** through out-of-phase ac signals (χ″_M_) that were detected at H_dc_ = 0 G. In this way, both **1** and **2** display single-molecule magnet (SMM) behavior, which is typical for all the members of this oxime-based family of Mn_6_ compounds [15,16,17,18,19,20,21,22,23,24,25,26,27]. The magnetic relaxation of **1** and **2** was investigated using out-of-phase (χ_M_″) ac susceptibilities vs. frequency (ν/Hz) plots and out-of-phase (χ_M_″) ac susceptibilities vs. temperature (T/K) plots, which are given in Figure 7 and Figure 8, respectively. In the two types of ac plots, the data of each maximum in **1** show much higher intensity than those data of **2**. Nevertheless, a similar magnetic relaxation would be observed for **1** and **2**.

We have reported previous works dealing with systems where two Mn_6_ molecules coexist in the crystal structure and, hence, two clearly distinguishable χ_M_″ peaks are observed and associated with the two Mn_6_ molecules [21,24]. This fact is not detected in the ac experimental data of compound **1**.

In the insets of Figure 8, it is shown the ln(τ) vs. 1/*T* plots for **1** and **2**. In both compounds, the plotted data follows a straight line covering the ranges of approximately 0.27–0.38 (**1**) and 0.33–0.47 K^−1^ (**2**). These data were fitted through the same mechanism for the relaxation of magnetization, namely, Orbach, and following the Arrhenius equation or eqn (2), where *τ*_o_ is the pre-exponential factor, *τ* is the relaxation time, U_eff_ is the barrier to relaxation of the magnetization, and k_B_ is the Boltzmann constant. The fitted data were plotted in their respective insets (Figure 8). Once fitted the experimental data of **1** and **2** using a least-squares fit of eqn (2), these parameters were obtained: *τ*_o_ = 2.54(1) × 10^−10^ s and U_eff_ = 40.9(1) K [28.4(1) cm^−1^] for **1** and *τ*_o_ = 2.80(2) × 10^−10^ s and U_eff_ = 33.0(2) K [22.9(2) cm^−1^] for **2**.

*τ*^−1^ = *τ*_o_^−1^exp(−U_eff_/k_B_*T*) (2)

The *τ*_o_ values reported for **1** and **2** are in concordance with those earlier published for Mn_6_ SMMs with S = 4 [13,17], and their computed U_eff_ values fall into the range previously reported for this family of compounds [24.0 K (16.7 cm^−1^) < U_eff_ < 86.0 K (59.8 cm^−1^)] [15,16,17,18,19,20,21,22,23,24,25,26,27].

## 3. Materials and Methods

### 3.1. Preparation of the Complexes

#### 3.1.1. Synthesis of **1**

MnCl_2_·4H_2_O (150 mg, 0.75 mmol) was poured with constant stirring to a solution generated by salicylamidoxime (114 mg, 0.75 mmol) and lipoic acid (350 mg, 1.7 mmol) in MeOH (25 mL), then NEt_3_ (500 µL, 0.9 mmol) was added. After a 2 h reaction, a dark green solution was formed along with a dark green microcrystalline solid of **1**, which was separated through filtration and washed up with ethyl ether. Yield ca. 70%. Crystals suitable for X-ray diffraction data collection were grown through a concentrated acetone solution of the microcrystalline solid with MeOH. These crystals present two crystallographically independent Mn_6_ complexes, one of them containing 2-cyanophenolate ligand which was generated by partial hydrolysis of salicylamidoxime during the recrystallization process. Elemental analysis was calculated (found) for the desolvated C_64_H_68_O_24_N_12_S_4_Mn_6_ compound: C, 41.6 (42.0); H, 3.7 (3.9); N, 9.1 (9.0)%. Selected IR data (in KBr/cm^−1^): peaks were observed at 3420 (s), 3312 (s), 1615 (vs), 1564 (m), 1530 (s), 1481 (w), 1441 (s), 1385 (w), 1310 (w), 1250 (w), 1143 (vw), 1020 (m), 908 (s), 880 (m), 750 (w), 681 (m), 640 (w), 578 (vw).

#### 3.1.2. Synthesis of **2**

Mn(NO_3_)_2_·4H_2_O (0.173 g, 0.688 mmol) was dissolved with continuous stirring in EtOH (20 mL), then salicylamidoxime (0.103 g, 0.670 mmol) was added, followed by lipoic acid (350 g, 1.7 mmol) and NEt_3_ (0.1 mL, 0.72 mmol). The resulting dark green solution was stirred for 1 h and was evaporated at room temperature. Crystals of **2** were obtained in 5 days and were suitable for X-ray diffraction. Yield: 55%. Elemental analysis was calculated (found) for the desolvated C_70_H_96_O_24_N_12_S_4_Mn_6_ compound: C, 43.2 (42.9); H, 5.0 (4.8); N, 8.6 (9.0)%. Selected IR data (in KBr/cm^−1^): 3426 (s), 3314 (s), 1614 (vs), 1564 (m), 1529 (s), 1483 (w), 1442 (s), 1384 (w), 1311 (w), 1251 (w), 1147 (vw), 1022 (m), 910 (s), 881 (m), 755 (w), 683 (m), 642 (w), 580 (vw).

### 3.2. X-ray Data Collection and Structure Refinement

X-ray diffraction data collection on single crystals of dimensions 0.13 × 0.04 × 0.03 (**1**) and 0.13 × 0.06 × 0.05 mm^3^ (**2**) were collected on a Bruker D8 Venture diffractometer with PHOTON II detector and by using monochromatized Mo-K_α_ radiation (λ = 0.71073 Å). Crystal parameters and refinement results for **1** and **2** are summarized in Table 1. The structures were solved by standard direct methods and subsequently completed by Fourier recycling using the SHELXTL [39] software packages and refined by the full-matrix least-squares refinements based on F^2^ with all observed reflections. The final graphical manipulations were performed with the Diamond [40] and CrystalMaker [41] programs. CCDC 2,253,180 and 2,253,181 for **1** and **2**, respectively.

### 3.3. Physical Measurements

Elemental analyses (C, H, N) were carried out on an Elemental Analyzer CE Instruments CHNS1100 in the Central Service for the Support to Experimental Research (SCSIE) at the University of Valencia. Infrared spectra (IR) of **1** and **2** were recorded with a PerkinElmer Spectrum 65 FT-IR spectrometer in the 4000–400 cm^−1^ region. Variable-temperature, solid-state (dc and ac) magnetic susceptibility data were measured on Quantum Design MPMS-XL SQUID and Physical Property Measurement System (PPMS) magnetometers. Experimental magnetic data were corrected for the diamagnetic contributions of both the sample holder and the eicosene. Further, the molecular diamagnetic contribution for each compound was corrected through the tabulated Pascal’s constants [42].

## 4. Conclusions

In conclusion, the preparation, the structural study and the magnetic features of two novel Mn_6_ compounds based on oxime and lipoate ligands have been reported. The investigation of the magnetic properties of these Mn_6_ systems through dc magnetic susceptibility measurements reveals a very similar magnetic behavior, with both compounds exhibiting both ferromagnetic and antiferromagnetic exchange among Mn(III) metal ions. In addition, ac magnetic susceptibility studies show the slow relaxation of magnetization at H_dc_ = 0 G, with these features showing the occurrence of the single-molecule magnet (SMM) phenomenon in both manganese compounds.

The relative stability and structural features of these two Mn(III) systems make them suitable SMMs to be studied on devices in the field of molecular spintronics. Indeed, we believe that our new lipoate-based complexes could be adequate systems to be grafted on surfaces or connected to junction devices, as previously done with similar Mn(III) systems functionalized with sulfur-based groups. This investigation is underway.

## Figures and Tables

**Table 1:** Summary of the crystal data and structure refinement parameters for **1** and **2.**

Compound	1	2
CCDC	2,253,180	2,253,181
Formula	C_136_H_182_N_26_O_56_S_4_Mn_12_	C_72_H_104_N_12_O_27_S_4_Mn_6_
Fw/g mol^−1^	3864.58	2027.55
Crystal system	Triclinic	Monoclinic
Space group	*P*ī	*C*2/*c*
*a*/Å	13.048(1)	20.482(1)
*b*/Å	13.185(1)	17.701(1)
*c*/Å	27.654(2)	24.934(1)
α/°	91.06(1)	90
β/°	99.89(1)	103.91(1)
γ/°	115.57(1)	90
*V*/Å^3^	4204.6(5)	8774.5(4)
*Z*	1	4
*D*_c_/g cm^−3^	1.526	1.535
μ(Mo-K_α_)/mm^−1^	1.008	1.014
*F*(000)	1992	4200
Goodness-of-fit on *F*^2^	1.037	1.126
*R*_1_ [*I* > 2σ(*I*)]/all data	0.0900/0.1569	0.0938/0.1145
w*R*_2_ [*I* > 2σ(*I*)]/all data	0.2042/0.2443	0.2328/0.2447

## Data Availability

The raw data that support the findings of this study are available from the corresponding author upon reasonable request.

## Supplementary Materials

The following supporting information can be downloaded at: https://www.mdpi.com/article/10.3390/ijms24108645/s1.
