# Supplementary material for: Lipoic Acid-Functionalized Hexanuclear Manganese(III) Nanomagnets Suitable for Surface Grafting
Source: Int J Mol Sci. 2023 May 12;24(10):8645. doi: 10.3390/ijms24108645 (PMC10218575; doi:10.3390/ijms24108645)
Supplement: Supplementary file 1 [file ijms-24-08645-s001.zip › ijms-2350141-supplementary.pdf]

Supplementary Information (SI)

Lipoic Acid-Functionalized Hexanuclear Manganese(III) Nanomagnets Suitable for Surface Grafting

Marta Orts-Arroyo, Carlos Rojas-Dotti, Nicolás Moliner and José Martínez-Lillo *

Departament de Química Inorgànica, Instituto de Ciencia Molecular (ICMol), Universitat de València, c/Catedrático José Beltrán 2, 46980 Paterna, Spain; marta.orts-arroyo@uv.es (M.O.-A.); carlos.rojas@uv.es (C.R.-D.); fernando.moliner@uv.es (N.M.)

*Correspondence: f.jose.martinez@uv.es; Tel.: +34-9635-44460

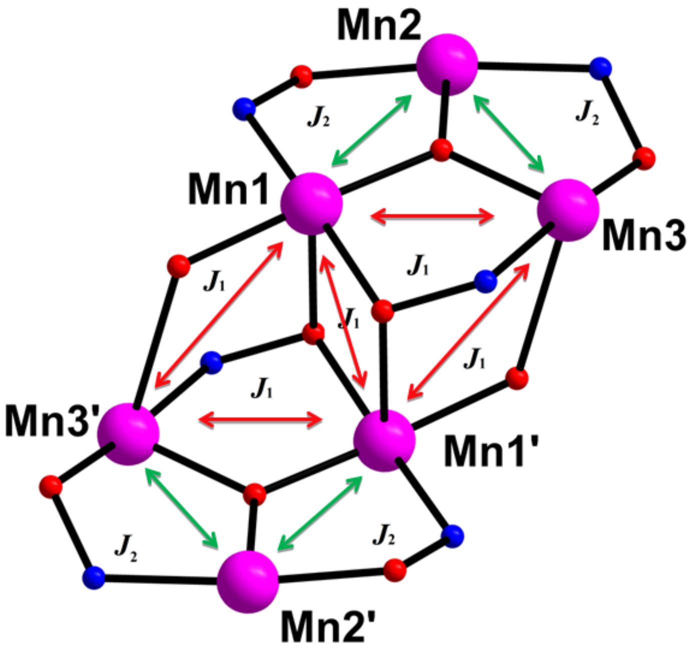

Figure S1. The 2-*J* coupling exchange model used to fit the experimental magnetic data of **1** and **2**.

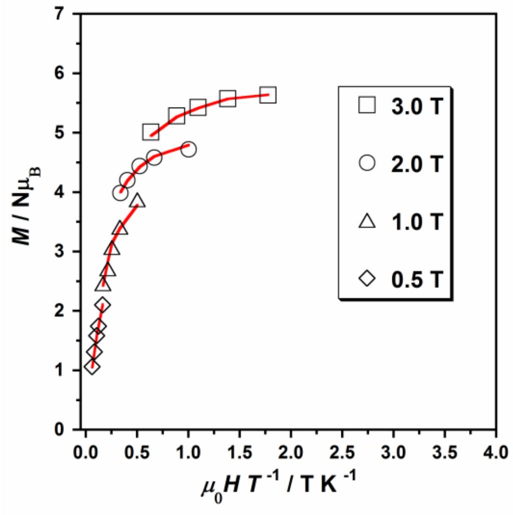

Figure S2. Plot of the reduced magnetization ($M/N\mu_B$ vs $\mu_o H/T$) at the indicated dc fields and temperatures 2–7 K for **1**. The solid lines represent the best fit of the experimental data [S = 4, g = 1.99 and D = -0.75 cm^{-1}].

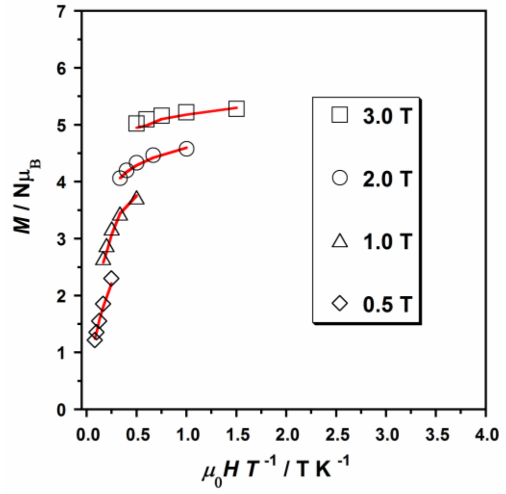

Figure S3. Plot of the reduced magnetization ($M/N\mu_B$ vs $\mu_o H/T$) at the indicated dc fields and temperatures 2–7 K for **2**. The solid lines represent the best fit of the experimental data [S = 4, g = 1.99 and D = -0.92 cm^{-1}].
